# A socio-ecological approach to physical activity interventions in childcare: a systematic review

**DOI:** 10.1186/1479-5868-11-22

**Published:** 2014-02-22

**Authors:** Marjo Anette Kristiina Mehtälä, Arja Kaarina Sääkslahti, Mari Elina Inkinen, Marita Eija Helena Poskiparta

**Affiliations:** 1 Department of Health Sciences, University of Jyväskylä, Keskussairaalantie 4, Jyväskylä 40014, Finland; 2 Department of Sport Sciences, University of Jyväskylä, Jyväskylä, Finland

**Keywords:** Physical activity, Intervention, Children, Childcare, Socio-ecological model

## Abstract

The promotion of physical activity (PA) in young children requires effective interventions. This article reviews the evidence on PA interventions in childcare by applying a socio-ecological approach. A computer-based literature search for intervention studies aimed at increasing children’s PA levels was run across four databases: SPORTDiscus, ISI Web of Science, PsycINFO and ERIC. The participants had to be in childcare, aged 2-6-year-old, and their pre- and post- intervention PA levels measured. Selection was restricted to peer-reviewed publications and to studies conducted in childcare settings. Twenty-three studies met the inclusion criteria and their methodological quality was assessed. Seven studies exhibited high methodological quality; twelve were rated as moderate and four low. The effectiveness of the interventions was determined according to the post-intervention behavioral changes reported in children’s PA. Fourteen studies found increases in PA levels or reductions in sedentary time, although the changes were modest. The data remain too limited to allow firm conclusions to be drawn on the effectiveness of the components mediating PA interventions, although PA-specific in-service teacher training seems a potential strategy. The findings of this review indicate that children’s PA remained low and did not approach the 180 min/day criteria. It may be that more intensive multilevel and multicomponent interventions based on a comprehensive model are needed.

## Introduction

Physical activity (PA) guidelines for childcare children (2–6 year olds) vary, stipulating between at least 2 hours and, more recently, at least 3 hours of PA daily [1-4]. Most children do not meet any of the current guidelines, and their PA levels have been reported to be very low [5,6].

Health behavior habits in childhood tend to track into adulthood. Findings of reviews have indicated evidence of moderate tracking during early childhood and from early childhood to middle childhood, and low to moderate tracking from childhood to adulthood for PA [7,8]. Increased or higher PA in early childhood reduces the risk for being overweight and is associated with improved motor skill development, psychosocial health, and cardiometabolic health indicators [9,10]. In contrast, a sedentary lifestyle is associated with greater risk factors for chronic diseases such as diabetes mellitus and coronary heart disease [1]. Therefore promotion of PA should begin already during early childhood.

Studies have reported low levels of PA among children attending childcare [11,12]. However, variation has been found in the amount and intensity of PA between children in different centers. This variation gives us reason to believe that factors facilitating and inhibiting PA exist in the childcare environment [13]. In addition, as most children attend childcare and the children’s families are in constant contact with them [14,15], centers offer an important intervention opportunity [16].

The most successful public health programs have been based on an understanding of health behaviors and the contexts in which they occur [17]. The socio-ecological approach emphasizes that health promotion should focus not only on intrapersonal behavioral factors but also on the multiple-level factors that influence the specific behavior in question. The socio-ecological model thus focuses on the interrelationships between individuals and the social, physical and policy environment [18].

**Figure 1:**
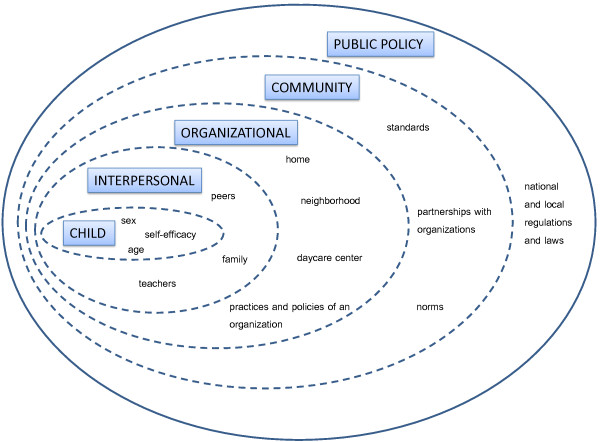
**Socio-ecological model.** Adapted from McLeroy KR, Bibeau D, Steckler A, Glanz K. An ecological perspective on health promotion programs. *Health Educ Q* 1988, 15:351–377.

It has been suggested that a comprehensive approach, such as that offered by the socio-ecological model [see Figure 1], is essential for examining the multiple level factors that might be determinants of PA [19]. The model helps us to identify opportunities to promote PA by recognizing the individual (e.g. sex, beliefs, and attitudes), behavioral (sedentary and active time), and social environmental (family, teachers, peers) and physical environmental (e.g. availability of PA equipment and facilities) factors that may influence one’s ability to be sufficiently physically active [20,21].

As far as we know, only one review has examined PA interventions in childcare-age children in center-based settings (e.g. daycare centers, preschools or nurseries) [22]. Although in their review Ward et al. [22] divided PA intervention studies into two behavior settings – curriculum and environment – none of the previous reviews have fully applied the socio-ecological approach. Furthermore, the promotion of PA among childcare children had barely begun at that time, and consequently more studies have since been published. In the present review, we examine the PA component of interventions designed to promote PA in children. The aim, utilizing the socio-ecological approach, is to identify potential targets (modifiable intrapersonal, interpersonal, organizational, community and/or policy level factors) and leverages for change in childcare-aged children’s PA promotion programs in a childcare setting [21].

## Methods

For the purposes of this review, the term “childcare” refers to center-based care for 2-to 6-year-old children. ‘Center’ includes facilities whether public- or private-sector operated, such as preschools, kindergartens, nurseries and Head Start centers. “Physical activity (PA)” is any bodily movement produced by skeletal muscles that requires greater energy expenditure than resting. “Exercise” is a subset of PA that is planned, structured, and often repetitive [23].

A computer-based literature search was carried out in May 2013. The search was conducted in four databases: SPORTDiscus, ISI Web of Science, PsycINFO and ERIC. A list of search terms and keywords, modified to reflect the aim of this review, was constructed on the basis of existing reviews [22,24]. The search strategy focused on free-text keywords referring to PA, childcare setting, child and intervention [see Table 1]. Previous reviews were also analyzed to search for potential studies missed in the initial literature searches.

**Table 1:** Detailed search terms

**Database**	**Search terms**
SPORTDiscus	SU (physical activit* OR physical fitness OR motor skills OR exercise OR physical education) AND SU (preschool OR child* OR nursery OR kindergarten OR child care OR daycare center) AND SU (intervention OR prevention Or promotion)
	Limiters - Peer Reviewed; Language: English
	Number of references retrieved: 446
Web of science	(TS = (physical activit* OR exercise OR motor skill* OR physical fitness OR physical education) AND TS = (preschool OR nursery OR kindergarten OR child* OR child care OR daycare center) AND TS = (intervention OR prevention OR promotion) NOT TS = (disabilit* OR disorder* OR cancer OR violence OR abuse OR clinical OR cerebral palsy OR Down syndrome OR patient OR asthma OR alcohol OR injur* OR HIV OR AIDS OR surger* OR delayed OR smoking OR Sickle Cell OR retardation OR pregnancy OR aggress*)) AND Language = (English) AND Document Types = (Article)
	Number of references retrieved. 4186
PsycINFO	su(physical activit* OR physical education OR physical fitness OR motor skills OR exercise) AND su(child* OR preschool OR nursery OR daycare center OR child care OR kindergarten) AND su(intervention OR prevention OR promotion) AND (peer(yes) AND la.exact(“English”) AND age.exact(“Preschool Age (2–5 Yrs)” OR “Childhood (birth-12 Yrs)”) AND po.exact(“human”))
	Number of references retrieved: 1017
ERIC	su(physical activit* OR physical fitness OR physical education OR motor skills OR exercise) AND su(preschool OR daycare center OR child care OR child* OR nursery OR kindergarten) AND su(intervention OR prevention OR promotion) AND (peer(yes) AND la.exact(“English”) AND lv(“early childhood education” OR “kindergarten” OR “preschool education”))
	Number of references retrieved: 108

Note: *Truncation = the word inflection suffixes have been left out of the string.

The inclusion criteria were: (a) 2-6-year-old children with no diagnosed diseases or health problems; (b) at least one intervention component of the study was targeted at increasing children’s PA; (c) children’s PA levels were measured (proxy-reported or objectively measured); (d) the study was carried out in a childcare setting (daycare center, preschool, nursery, long daycare center); and (e) the study had been peer-reviewed and published in English. We chose to include only center-based interventions and not e.g. family childcare, as most childcare-aged children attend a childcare center [14,15]. In addition to RCTs, the search also included quasi-experimental, before/after, pilot and feasibility study designs, as the reviewers were aware that the research area was rather new and that confining the search to RCTs was unlikely to yield more than few candidate studies. No limit was set on date of publication.

**Figure 2:**
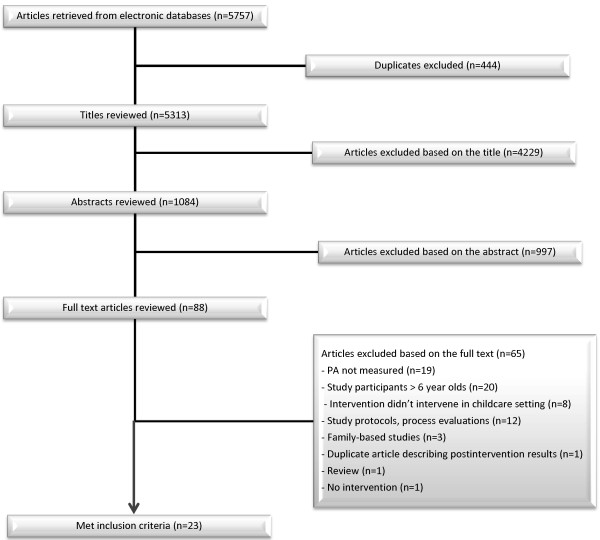
Flow diagram of article selection.

Two reviewers independently reviewed the titles obtained from the initial searches. When necessary, abstracts were read and, finally, the entire paper. The reviewers independently assessed the full text of the retrieved articles and when opinions differed, consensus was reached through discussion. The percentage of inter-reviewer agreement on the articles to be included was calculated by the formula [number of included articles/(number of included articles + number of articles under discussion)] × 100 [Figure 2].

Two reviewers evaluated the included studies for selection bias, study design, confounders, blinding, data collection methods, withdrawals and dropouts, analysis and intervention integrity, using the validated eight-component *Quality Assessment Tool for Quantitative Studies*[25]. Each of the components, except for analysis and intervention integrity, was rated as weak, moderate, or strong. The components were assessed on the basis of the manual accompanying the quality tool [25]. In this review, we used the *quality rating scale* described in Hesketh and Campbell’s [24] systematic review. Where a component was not described, the rating weak was given, except for blinding, where the rating moderate was given. The overall study rating was weak if two or more of the six components were weak, moderate if less than four ratings were strong and one rating was weak, and strong if at least four ratings were strong and no ratings were weak [24]. The component quality ratings for selection bias, study design, confounders, blinding, data collection methods, withdrawals and dropouts given by the two reviewers were then compared, and ‘percentage inter-rater agreement’ calculated by the formula [(number of articles included in the review × number of quality tool components - number of disagree ratings/(number of articles included in the review × number of quality tool components)] × 100.

## Results

After duplicates were excluded a total of 5 313 publications were retrieved from the database searches. After screening the titles and abstracts of the publications, 88 publications were considered potentially eligible. Based on the full text, 65 publications were excluded from the final review. The most frequent reasons for exclusion were that the children’s PA was not measured (n = 19), or that the study participants were over age 6 (n = 20). In eight publications, the intervention was not implemented in a childcare setting; in twelve, study protocols or process evaluations were reported without PA outcome results; in three, the study was family-based; one was a duplicate describing postintervention results; one was a review, and one was not an intervention study [see Figure 2]. All in all, twenty-three intervention studies targeted at promoting childcare-age children’s PA met the inclusion criteria and were assessed for their methodological quality.

The percentage of inter-reviewer agreement on the inclusion of articles was 85%. Discrepancies over inclusion occurred in four cases. It was decided to exclude these articles from the final review as two of them were family-based studies in which the childcare centers only served as settings for the recruitment and randomization of the study participants [26,27]. In the other two studies, the children were over age 6 [28,29]. The percentage of inter-rater agreement for methodological quality was 82%. All the disagreements in the quality assessments were due to differences in interpretation of the criteria. Detailed descriptions of the included studies are given in an additional file [see Additional file 1].

### Characteristics of the included studies

#### Quality

High methodological quality was exhibited by seven studies [30-36]; three of which also had a large sample size [32,34,35]. Twelve studies were rated moderate in quality [37-48]. Of the remaining four studies, with low quality ratings, two focused on exploring children’s PA patterns during a short intervention [49,50] and two modified the outdoor play environment [51,52]. The study design section was assessed as strong in fifteen of the cluster-randomized controlled trials (RCT) [30-42,44,47] and as moderate in one quasi-experimental study [45]. The designs of the remaining studies were rated as weak, as they were either case–control studies [50], before/after studies [43,48,49,52] or lacked a control group [46,51]. Eight studies were reported to be pilot, preliminary or feasibility studies [30,37,38,44,48,49,51,52]. Seven studies focused on subsamples of childcare children (i.e. low socioecomic or migrant background or specific ethnicity) [30,35,37,39,41,42,46]. Nine studies reported that their designs were grounded in behavior theory [34,35,39,41,42,45-48]. Only three of the RCTs reported on whether the method of random allocation was concealed [35,44,47] and five on whether the outcome assessor (s) was blinded [31,35,41,44,47]. The retention rate was very high (80-100%) in 18 studies [30,32-39,42-45,47-50,52] and good (60-79%) in 4 studies [31,40-42]. In one study using cross-sectional samples, this item was not applicable [51].

#### Country

Seventeen studies were carried out in the United States [30,31,34,36-43,45,46,48-51]. Four studies were carried out in Europe (two in Belgium [32,52], one in Switzerland [35], one in Scotland [47]), one in Australia [44] and one in the Middle East (Israel) [33].

#### Program length

The duration of the intervention ranged from two days to 12 months and program length varied from 4 days to 24 months. In three studies, the intervention was six months or over [30,31,35], in eight studies 14–24 weeks [33,34,40-42,44,45,47] and in the remaining studies less than 14 weeks [32,36-39,43,46,48-52]. Only four studies had a follow-up of 6 months or longer [31,41,42,47]. Structured physical activities formed one component in 18 interventions [30,31,33-36,38-40,42,44-48]. Structured activities were arranged every day (duration 10–30 min) in seven interventions [30,31,33,36,39,45,48], 2–4 times per week (duration 15–45 min) in eight interventions [34,35,38,40-42,44,47], and structured sessions were compared to free play sessions in three interventions [46,49,50].

#### PA assessment

The method used to assess children’s PA depended on the aim of the study. Whole-day assessments were conducted in eleven studies [30,31,33-35,37,38,40,47,50],[52]. Assessments were made during childcare attendance in five studies [36,39,51], during a recess in four studies [32,43,46,49], at active points in two studies [45,48] and during the time after childcare in two studies [41,42]. Children’s PA was assessed using accelerometers in thirteen studies [30-32,34,35,37-39,43,44,47,52], pedometers in two studies [33,40], heart rate monitors in two studies [46,50], direct observation in three studies [45,48,49], proxy reports in two studies [41,42] and both accelerometers and direct observation in three studies [36,43,51]. The accelerometer data in the studies included in this review varied widely: epoch length ranged from 5 s to 1 min, although 15 s was most common (71%), while the number of days spent wearing an accelerometer ranged from 1 day to 10 consecutive days, and five different cut-points were used [53-56].

#### Levels of influence

Seven studies targeted only the childcare environment [31,32,37,43,50-52] and one study only the teacher [49]; two targeted the both childcare environment and the teacher [36,38], and four also targeted the child [30,33,39,44]. Two studies targeted the childcare and home environment, the teacher, the parent, and the child [45,47], and four studies targeted the childcare environment, the teacher, the parent and the child [34,35,40,48]. Two studies targeted the childcare environment, the parent and the child [41,42]. Twelve studies were conducted at three levels of influence: intrapersonal (IND), interpersonal (INT) and organizational (ORG) [30,33-35,39-42,44,45,47,48]. Three studies were conducted at two levels: ORG and INT [36,38] or ORG and IND levels [46]. The remaining eight studies were conducted at only one level: ORG [31,32,37,43,50-52] or INT [49]. None of the interventions reviewed here were conducted at more than three levels of influence, and none at the community and/or policy level.

#### Effects on PA

In seven of the twenty-three studies, the main focus was other than PA (e.g. BMI, motor skills, bone health) and in two of these studies the children’s PA levels increased significantly [31,34]. The remaining studies focused on increasing children’s PA levels, and significant increases in PA and/or decreases in sedentary levels were reported in twelve studies [30,33,36,38,39,43,44,46],[49-52].

All in all, significant changes in children’s PA levels were reported in fourteen (61%) intervention studies, five of which were rated high in methodological quality [30,31,33-35], five as moderate [38,39,43,44,46], and four as low [49-52]. Five studies found no significant changes in children’s PA levels, but found a positive intervention-based trend in PA or significant positive changes in aerobic fitness or motor skills, i.e. in factors associated with PA [35,40,45,47,48].

Table 2 summarizes the evidence on the effectiveness of the interventions included in this review. The findings of the studies are stratified by the intervention strategies used to promote children’s PA. Visual inspection of Table 2 indicates that the high quality studies were more likely to report a significant increase in children’s PA than the lower quality studies, except when they used playground or play-time modifications as a PA promotion strategy. A significant increase in children’s PA was reported by a greater percentage of non-theory-based than theory-based studies (86% vs. 33%). Of the nine theory-based studies, a significant increase in PA was observed in three [34,39,46], of which PA was the primary outcome in two [39,46]. PA was a secondary outcome in five theory-based interventions, of which in four no significant increase in PA was observed [41,42,45,47].

**Table 2:** PA effects of included studies stratified by intervention strategies

	**Studies**^**1**^					
	**High quality multi-level theory-based**		**High quality**		**Lower quality**^**2**^	
**Strategy**	**Significant**	**Non-significant**	**Significant**	**Non-significant**	**Significant**	**Non-significant**
***Organizational level***						
**Structured PA**						
Every day (10-30 min)			Alhassan [30]		Annesi [39]^3^	Winter & Sass [45]^3^
			Binkley & Specker [31]			Sharma [48]^3^
			Eliakim [33]			
			Trost [36]			
2-4 per week (15-45 min)	Fitzgibbon [34]	Puder [35]	Fitzgibbon [34]^3^	Puder [35]^3^	Alhassan [38]	Bellows [40]
					Jones [44]	Fitzgibbon [41]^3^
						Fitzgibbon [42]^3^
						Reilly [47]^3^
Compared to free play					Parish [46]^3^	
					Brown [49]	
					Deal [50]	
**Playground/-time modifications**		Puder [35]		Cardon [32]	Hannon & Brown [43]	Alhassan [37]
				Puder [35]^3^	Nicaise [51]	
					Van Cauwenberghe [52]	
***Interpersonal level***						
**Teacher involvement**	Fitzgibbon [34]	Puder [35]	Alhassan [30]	Puder [35]^3^	Alhassan [38]	Bellows [40]
			Eliakim 2007 [33]		Annesi 2013 [39]^3^	Winter & Sass 2011 [45]^3^
			Fitzgibbon 2011 [34]^3^		Jones 2011 [44]	Reilly 2006 [47]^3^
			Trost 2008 [36]		Parish 2007 [46]^3^	Sharma 2011 [48]^3^
**Parental involvement**	Fitzgibbon 2011 [34]	Puder 2011 [35]	Fitzgibbon 2011 [34]^3^	Puder 2011 [35]^3^		Bellows 2013 [40]
						Fitzgibbon 2005 [41]^3^
						Fitzgibbon 2006 [42]^3^
						Winter & Sass 2011 [45]^3^
						Reilly 2006 [47]^3^
						Sharma 2011 [48]^3^
***Intrapersonal level***						
**Knowledge, beliefs, motor skills, aerobic fitness, self-efficacy**	Fitzgibbon 2011 [34]	Puder 2011 [35]	Alhassan 2012 [30]	Puder 2011 [35]^3^	Annesi 2013 [39]^3^	Fitzgibbon 2005 [41]^3^
			Eliakim 2007 [33]		Jones 2011 [44]	Fitzgibbon 2006 [42]^3^
			Fitzgibbon 2011 [34]^3^		Parish 2007 [46]^3^	Winter & Sass 2011 [45]^3^
						Reilly 2006 [47]^3^
						Sharma 2011 [48]^3^

Notes: ^1^Named by first author, ^2^Moderate and low quality studies, ^3^Theory-based intervention.

### Intervention strategies

#### Intrapersonal level

When children were given the time and opportunity to practice fundamental motor skills, their motor skills performance improved [30,35,44]. In the two of these studies where PA was the primary outcome, a significant increase in children’s PA or a reduction in sedentary time was also observed [30,44]. When PA was a secondary outcome, improvement in motor skills, but not a significant increase in PA, was observed [35,45,47].

More playground space was associated with higher post-test PA levels in boys than girls, independent of the intervention condition [32]. The authors suggested that the reason for this was that boys commonly engage in more sports-like activities than girls. Structured activities resembling those children prefer and normally engage in when they are active encouraged greater participation in PA [33,49].

However, in non-competitive environments no sex differences in PA were observed [46,50], and when playground density was lowered by scheduling more recesses, girls benefited even more during recess than boys. No sex differences were observed in an analysis of whole-day PA data [52].

#### Interpersonal level

Of the high quality studies, five included a PA-related teacher training intervention component [30,33-36]. All except one of these studies [35] found significant positive changes in PA. When encouraged and guided by adults, as compared to non-intervention periods, children’s MVPA increased [46,49]. Children were also more enthusiastic and moved vigorously when teachers joined in with them in activities such as tag [46].

In eight of the studies that had a home component, i.e. parental involvement in the intervention, only one reported a significant increase in PA [34]. One study was a pilot [48]. Two studies by Fitzgibbons et al. (2005; 2006) used parent-proxies [41,42]. In a similar, but teacher-delivered, intervention, significant increases in objectively measured PA were observed [34]. Significant improvements were observed in children’s motor skills, but were not accompanied by any significant increase in PA [35,40,45,47].

#### Organizational level

Provision of new play equipment momentarily increased children’s PA levels [43], but in the longer term an activity-friendly playground was insufficient to elevate PA levels [32]. More space per child [52] and a substantial amount of an additional structured [38], but not free, outdoor play time [37] were associated with more PA during recess. Specific playground features, such as an open space grass area, a grass hill, and a looping cycle path, were observed to associate with greater MVPA [51].

Structured PA was used as a PA promotion strategy in most of the interventions included in this review. Promising results were observed in these studies, all of which used relatively brief PA sessions [36,39,49,50] at intervals across the day [33]. By varying the activities to be engaged in over time, children were able to maintain MVPA [50]. Visual inspection of Table 2 indicates that the intervention was more likely to be effective when structured or when adult-led activities are arranged every day rather than less frequently.

## Discussion

To our knowledge, this is the first review that has attempted to identify potential strategies for increasing PA at each level of influence of the socio-ecological model in the case of childcare children (2-6-year-old). Despite the increasing interest in the promotion of PA among childcare children, the number of published studies remains low. Hence the inclusion of lower as well as high quality studies in this review enabled us to view things more comprehensively than would have been possible had the analysis been restricted to RCTs. However, the findings from the interventions were mixed and the level of evidence inconclusive.

Among the studies reviewed here, in addition to structured PA, the use of PA-specific in-service teacher training as intervention strategy was potentially fruitful [see Table 2]. Moreover, teachers’ experience and personal characteristics may play an important role in increasing PA among childcare children [34,44,57,58]. However, due to the lack of mediating analysis to assess possible causal pathways between these strategies and increased PA in childcare children [30,33,34,36], this task remains for future studies.

According to the socio-ecological model, children interact with others in their most immediate learning and development environment. This approach identifies the family as the most influential and proximal system. It also recognizes the importance of partnership between families and childcare [20,59]. Together, parents and teachers have the best knowledge of the barriers children encounter when engaging in their routine daily PA and the potential that exists for PA in both the home and daycare contexts [60,61]. Given the positive associations found earlier between parental support and children’s PA [60], parental support was expected to be a potential strategy. However, based on the present review, the influence of parents on their childcare children’s PA remains unclear. In the eight studies with a parental component, only one high quality intervention succeeded in significantly increasing PA. It may be that families need to be more strongly committed to the intervention, and that merely giving parents knowledge or materials is not enough in a center-based intervention [47].

On the other hand, the non-significant results of the studies on parental influence on children’s PA included in this review should be interpreted with caution. More methodologically sound research is needed in this area. Two studies by Fitzgibbons et al. (2005; 2006) used parent-proxies, which could have masked significant increases in PA [41,42]. The study by Reilly et al. [47] was methodologically promising, but only potentially effective. The authors suggested that the home component was not intensive enough [47]. In a childcare pilot, with rather intensive parent involvement (parent tip-sheets twice a week), parent involvement and school-parent communication during the intervention were found to be very important. Unfortunately, the study lacked the power to detect possible increases in children’s PA. Also fewer PA opportunities were scheduled postintervention than preintervention revealing that the study was not implemented as planned [48]. Some of the studies with a home component limited their assessment to childcare attendance only [45,48], omitting the possible impact of parental social support [26], and leaving a question mark over activity levels during time outside childcare.

In this review, intervention strategies extending to the community and policy levels of influence are considered to be large scale, as they deliver and assess interventions broadly and are also highly visible, reaching larger numbers of people (see Figure 1, [17]). In the present review, interventions of this kind were lacking. However, from a socio-ecological perspective and what can be inferred from the studies included in this review, future research might usefully focus on the upper levels of the model in seeking ways to lower the barriers to increased child engagement in PA. We could make an effort to influence the overall culture of childcare centers, and especially the status of PA in them. Teachers’ cultural beliefs about play and learning are translated into actions which, in turn, influence children’s play behavior (i.e. PA) [62]. Copeland et al. [58] concluded that policies of childcare concerning children’s safety and school readiness may hinder children’s physical development [63]. The question is could we enhance children’s PA by changing environmental policies without jeopardizing their safety and school readiness? Intervention at the community level, while requiring a lot of resources, may eventually prove a sustainable and a cost-effective strategy [64,65]. Maintaining changes in health behavior is important, and to attain this goal calls, in particular, for long-term and post-intervention studies. Based on the lack of robust studies and conflicting results to date, further exploration of community interventions is warranted.

In this review only four studies had a follow-up of 6 months or longer [31,41,42,47]. More long-term evaluations are needed. Despite being feasible and highly acceptable to both teachers and children, teachers may lose the motivation to continue with a program postintervention [44]. Challenges reported by teachers in incorporating PA into the childcare curriculum included the weather, which could limit outdoor time and opportunities for active play, and lack of a designated gym area, which could restrict indoor physical activities [48,49]. To be effective, intervention programs may need modification, and hence teachers should learn to customize the activity patterns of the program to fit their particular curriculum and physical environment [45]. A solution for the resource problem could be found in integrated PA programs [37,45], which have successfully been tested, although their long-term effects, e.g. on academic performance, have yet to be clarified.

The review findings also highlight other gaps in knowledge. First, only two high quality theory-based multilevel and multi-componential studies were included, and in both studies PA was not a primary outcome [34,35]. Based on this review, the evidence is consistent with either an increase or no increase in PA as a result of the theory-based multilevel intervention. Overall, in this review theoretical-based studies were not more effective than non-theoretical studies. Although non-theoretical interventions were relatively more effective, it should be noted that in most of the theory-based studies PA was not a primary outcome. Also the extent to which the theory cited was in fact used in the intervention is unclear [66]. Consequently, it is not possible to take a position on their superiority or inferiority compared to individual-level interventions or non-theoretical-based studies. More robust multilevel intervention research, which operates also at the community level, is needed before this can be attempted [67,68].

Second, even where the intervention studies reported significant increases in PA levels, the results were nevertheless modest and the children’s post-intervention activity levels remained below the current PA recommendations [1-4]. However, it should be remembered that while recommendations are based on the best currently available research evidence, the optimal amounts and intensity levels of PA for children’s healthy growth and development remain unclear [69]. Secondly, half of the studies included in this review only measured children’s PA during childcare attendance or recess, which fails to take into account possible PA outside of childcare day.

Third, in childcare settings, studies have focused on exploring outdoor playgrounds to the neglect of indoor facilities, despite the fact that children spend a considerable amount of time indoors during childcare day [70]. That there is a role for teachers in enhancing children’s PA seems obvious, but none of the reviewed studies looked at peer influence on children’s PA; this should be examined in the future.

### Strengths and limitations of the study

This study has limitations which must be taken into account when interpreting the results. First, the review process revealed that although interest in promoting children’s PA has increased in recent years (48% articles of the reviewed articles were published after 2011), research in this area remains scarce. Second, it is possible that potential articles were missed due to the search strategies and criteria used; only studies published in English were included, leaving potential studies written in other languages out of account. Third, the fact that measuring children’s PA is a complex task [71], may have also affected study results and hence also the results of the present review. Future studies need to utilize valid and reliable methods of measuring PA [72]. Fourth, it should be noted that several studies in this review focused on subsamples of childcare children (i.e. low socioecomic or migrant background or specific ethnicity) [30,35,37,39,41,42,46]. When the study sample represents only a minority of the population generalizing their results to other groups must be done with caution.

Finally, we found that identifying studies with high methodological quality was challenging because of deficiencies in the reporting of studies. Concealment and blinding were reported in only a few articles [30,34,40,43,46]. Intention to treat was mentioned clearly in only three studies [34,41,43], and exposure to the intervention often remained unclear. However, in most studies the outcome measurements used and intervention itself were clearly described.

A major strength of our review was that the databases of several different disciplines – exercise science, general, psychology and pedagogy – were searched, enabling a wider range of intervention types to be found. Second, an advantage of present review compared to reviews that have examined factors associated to PA was that all the included studies were longitudinal and focused on a narrow target population. Children of childcare-age are in a developmentally different stage than older children. In fact, only a few PA-associated variables have been found to be the same in children and adolescents [73]; thus, childcare-age children may respond differently, for example, from primary school children to an intervention [32]. Third, two researchers independently reviewed the article titles and abstracts to identify potentially relevant studies, and then assessed the quality of those included in the review.

## Conclusions

Our systematic review of studies of interventions designed to promote childcare children’s PA yielded very few high quality interventions. Only two of the seven multilevel and theory-based studies found significant changes in children’s PA levels, and only one of these was rated as high quality. Based on available data we found no evidence of effect of multi-component and theory-based interventions.

Although it is difficult to draw general conclusions based on the mixed results of the studies included in this review, the most effective intervention strategy seems to lie in the personal characteristics, and more specifically PA in-service training of teachers. Future studies should pay more attention to the PA training of teachers, offering them more tools for promoting the level of PA engaged in by children’s during their attendance at childcare. Long term follow-up studies are also required to assess the maintenance of behavioral changes.

## Competing interests

The authors declare that they have no competing interests.

## Authors’ contributions

MAKM conducted the database searches, reviewed titles, abstracts and full papers for inclusion and synthesis of the systematic review, performed the quality assessments of the included studies and wrote the manuscript. MEI and MEHP reviewed titles, abstracts and full papers for inclusion and performed the quality assessments of the included studies. MEHP and AKS reviewed and commented on all content and all drafts. All authors read and approved the final manuscript.

## Authors’ information

MAKM is a PhD student in the Department of Health Sciences, Faculty of Sport and Health Sciences, University of Jyväskylä, Finland. MEI is a project researcher in the Department of Health Sciences, Faculty of Sport and Health Sciences, University of Jyväskylä, Finland. AKS is a senior researcher in sport pedagogy in the Department of Sport Sciences, Faculty of Sport and Health Sciences, University of Jyväskylä, Finland. MEHP is a professor in the Department of Health Sciences, Faculty of Sport and Health Sciences, University of Jyväskylä, Finland.

## Supplementary Material

Additional file 1 **Summary of included studies.** Detailed descriptions of the included studies in this review. Click here for file
