# Supplementary material for: A socio-ecological approach to physical activity interventions in childcare: a systematic review
Source: Int J Behav Nutr Phys Act. 2014 Feb 22;11:22. doi: 10.1186/1479-5868-11-22 (PMC3936868; doi:10.1186/1479-5868-11-22)
Supplement: Additional file 1 — Summary of included studies. Detailed descriptions of the included studies in this review. [file 1479-5868-11-22-S1.pdf]

Additional 1 Summary of included studies								
**Study**	**Quality rating**	**Sample**	**Intervention design *(reported theory)***	**Primary outcomes**	**Description of PA component**	**PA measurements**	**Effect on PA**	**Level of influence and targeted factor**
Alhassan et al. 2007, USA [32]	Moderate	33 Latino children, 3-5 yr, one Head Start center	Pilot RCT, unit: class	PA	Intervention group received an additional 30 min of outdoor free playtime in the morning and afternoon, for 2 consecutive d.	Accelerometer (ActiGraph) worn for 2 consecutive d at baseline and during the 2-d intervention. Valid data ≥10 h/d. Epoch length 30 s, cut-points by Sirard et al. (2005).	No significant differences in PA between the two groups in total PA time, at-school time or after-school time.	**Organizational**: outdoor playtime in center
Alhassan et al. 2012, USA, Project PLAY [25]	High	78 low-SES children, 3-5 yr, 2 preschool centers	Pilot RCT, unit: 8 classes randomized to LMS-PA (locomotor skills –oriented lesson plans) group and UF-PA (unstructured free playtime) group	Locomotor skills (LMS) and PA	30 min/d, 5 d/week for 6 months; LMS lessons consisted of 3-5 min low-intensity musical activity, 20 min LMS activities and 5 min extension activities. 8 h teacher training to learn proper execution of the program. In the control group intervention consisted of unstructured free play time.	Accelerometer (ActiGraph) worn for 7 consecutive d at baseline and at 6 months postintervention. Valid data ≥9h/d for ≥4 d. Epoch length 15 s, cut-points by Sirard et al. (2005).	LMS-PA group decreased significantly during-preschool (adj. mean difference -9.6%) and total day (adj. mean difference-9.3%) percent time spent in sedentary PA compared to baseline (p=.02; P=.01, respectively).	**Organizational:** structured playtime in preschools **Interpersonal:** teacher influence: social support **Intrapersonal:** locomotor skills

Additional file 1 Continued

Study	Quality rating	Sample	Intervention design *(reported theory)*	Primary outcomes	Description of PA component	PA measurements	Effect on PA	Level of influence and targeted factor
Alhassan et al. 2013, USA [33]	Moderate	67 children, 3-5 yr, 2 preschool programs, 8 preschool classes	Pilot RCT, unit: class, structured (STRUCT) and free outdoor playtime (CON) classrooms	PA levels	An additional 30-min of structured outdoor playtime (SPARK program) 3 d/week for 4 weeks. STRUCT teacher training 2x4 h and CONT teacher training 2 h. 12 lesson plans (3-5 min low-intensity activity, MVPA activity/game 20-25 min, low-moderate-intensity activity 3-5 min). Lessons implemented by trained research staff with the aid of classroom teachers.	Accelerometer (ActiGraph) worn for 7 consecutive d at baseline and during fourth week. Valid data ≥ 9h/d for ≥ 4 d. Epoch length 15 s, cut-points by Sirard et al. (2005).	Time spent in VPA significantly increased during the 30-min intervention for STRUCT compared to the CON (p=.04). Compared to CON, STRUCT spent significantly greater %time engaged in MVPA during total day (p=.03) and lower %time engaged in sedentary activity during the entire day and school (p=.01; p=.01). MVPA time increased 5.5 min and sedentary time decreased 9 min during the day.	**Organizational:** an additional structured outdoor playtime in center **Interpersonal:** teacher influence: social support
Annesi et al. 2013, USA, Start for Life [34]	Moderate	275 AA children, 3-5 yr, 32 preschools	Cluster RCT, unit: preschools, 21 treatment and 11 control schools (SCT)	PA during recess	Structured PA 30 min/d, 5 d/week for over 8 weeks; theory-based PA lessons consisted of 3-5 min warm up, 12 x rotated segments (1 min VPA, 30 s light PA, 1 min MVPA) and 3-4 min cool down with the behavioral skills training and self-regulation facilitating interspersed. In the control group intervention consisted of structured 30 min PA with highly variable activities. Teachers in the treatment group received an additional 4-hours training (Start for Life) + daily lessons plans.	Accelerometer (ActiGraph) worn 1 d at baseline and at after the 8-week intervention, analyzed wearing time 9.15 am - 2 pm Epoch length 15 s, cut-points by Pate et al. (2006).	Significant increases in MVPA and VPA among intervention group (58 and 38 min/week, respectively, assuming a school day of 7 h)	**Organizational:** structured PA in preschool **Interpersonal:** teachers influence: role modeling, social support **Intrapersonal:** cognitive-behavioral skills, self-regulation, self-efficacy, physical self-concept

Additional file 1 Continued								
**Study**	**Quality rating**	**Sample**	**Intervention design *(reported theory)***	**Primary outcomes**	**Description of PA component**	**PA measurements**	**Effect of PA**	**Level of influence and targeted factor**
Bellows et al. 2013, USA, Mighty Moves [35]	Moderate	263 children, 3-5 yr, 8 Head Starts	Cluster RCT, unit: preschool	Gross motor skills, PA and BMI	15-20 min/d, 4 d/week for 18 weeks, lessons (143 activities) focused on skills or a group of skills. Food Friend character with Mighty Move and super-power led children through the town of Healthadelphia®. PA lessons kit including e.g. teacher binder, PA equipment and parent materials. Intervention teachers received training on gross motor development and age-appropriate PA.	Pedometers (Walk4Life) worn 6 d (4 weekdays and 2 weekend days) at baseline and immediately after the intervention.	No significant differences in PA between groups.	**Organizational:** lessons and structured PA in preschool; **Interpersonal:** parental and teacher influence: social support **Intrapersonal:** knowledge, beliefs, attitude, motor skills
Binkley & Specker 2004, USA [26]	High	178 children, 3-5 yr, 11 childcare centers	Cluster RCT, follow-up phase, unit: centers, participants randomized to gross motor (GM) and fine motor (FM) activity groups	Leg bone mineral content and size	30 min/d, 5 d/week for 12 months; intervention consisted of jumping, hopping, skipping activities. Children in the control group received 30 min of fine motor activity.	Accelerometer (Actiwatch) worn for 48 h at baseline, 6 months, 12 months (post), 18 months and 24 months follow-up. Epoch length 1 min., VPA > 1000 cpm.	Significantly greater counts/d and higher % time in VPA in the GM than FM group at 12 and 18 months (p<.050), but not at 24 months.	**Organizational:** structured PA in childcare centers
Brown et al. 2009, USA [44]	Low	5 children, 4 yr, two preschools	Feasibility study, a single-case withdrawal. Comparison of children's PA during nonintervention and intervention outdoor play periods across day	MVPA	Two studies with similar teacher-implemented interventions. “Plan, Do and Review!” process focused on children’s MVPA. Two activities: Track Team consisted of jogging around the playground and Dance Party consisted of dancing to high-energy preschool music. Both activities were done with teachers and peers. Intervention and nonintervention procedures.	OSRAC-P used during 30 min outdoor play periods. 5 s-observation/25 s-recording intervals.	Increased % of intervals in MVPA on intervention days relative to nonintervention days. Visual inspection and analysis based on the graphs.	**Interpersonal:** teacher influence: teacher encouragement to PA

**Additional file 1 Continued**								
**Study**	**Quality rating**	**Sample**	**Intervention design *(reported theory)***	**Primary outcomes**	**Description of PA component**	**PA measurements**	**Effect of PA**	**Level of influence and targeted factor**
Cardon et al. 2009, Belgium [27]	High	583 children, 4-5 yr, 40 pre-schools	Cluster RCT, unit: preschools randomly assigned to 3 intervention and one control groups	PA during recess	4 groups: 1) play equipment was provided, 2) markings were painted on the playground, 3) play equipment was provided and markings were painted, 4) control.	Accelerometer (ActiGraph) worn during recess at baseline and 4 to 6 weeks after the intervention. Epoch length 15 s, cut-points by Sirard et al. (2005)	No significant increase in PA.	**Organizational:** outdoor play equipment and markings in preschools
Deal 1993, USA [45]	Low	33 children, 3–5 yr, move-ment program (n = 15) or com-munity preschool (n = 18)	Nonrandomized CT, explored PA patterns	PA	Movement program consisted of one 2-h session with each child getting one-on-one lessons led by a university student, series of 10-min activities included locomotors, manipulative stability or gymnastics skills, large muscle or climbing and fitness, along with a 20-min swim.	Heart rate measured with Polar Vantage XL worn 12 h. Heart rate readings recorded once every 60 s. To measure resting baseline heart rate watch worn 24 h, or at least overnight. Heart rate readings for program and home activities were grouped into 2 h-blocks of time. Valid data at least 2 h program activity and 2 h home activity.	Children's heart rate was significantly elevated from resting levels in a movement program (p<.001). During the movement program children spent 61% in MVPA, whereas during the same time at the daycare children spent 22% in MVPA.	**Organizational:** structured PA
Eliakim et al. 2007, Israel [28]	High	101 children, 5-6 yr, 4 preschool classes	Cluster RCT, unit: classes	Body weight, BMI, BMI percentile, body compositi on, PA and fitness	45 min/d divided into 15-min sessions, 6 d/week for 14 week. 2 d/week led by a professional youth coach and other d by preschool staff, used exercise circuit (indoors and outdoors) with endurance activities and some coordination and flexibility. Teachers were given instructions about intervention implementation.	Pedometers (Stepometers) worn at baseline and postintervention during school hours and after school hours for three weekdays.	Intervention group performed more steps overall (6927 vs. 5489) as well as during (3104 vs. 2365) and after school (3822 vs. 3123) compared to controls (p=.003; p=.001; p=.04, respectively).	**Organizational:** structured PA in preschools **Interpersonal:** teacher influence: social support **Intrapersonal:** knowledge, beliefs, attitude

Additional file 1 Continued								
**Study**	**Quality rating**	**Sample**	**Intervention design *(reported theory)***	**Primary outcomes**	**Description of PA component**	**PA measurements**	**Effect on PA**	**Level of influence and targeted factor**
Fitzgibbon et al. 2005, USA, Hip Hop to Health Jr. [36]	Moderate	409 AA children, 2-5 yr, 12 Head Start centers	Cluster RCT, unit: preschool *(SCT, SDT and TTM)*	BMI	40 min three times weekly for 14 week; 20-min lesson and 20-min physical activity component plus parent weekly newsletters.	Parents assessments of children's PA frequency and intensity pre-, post- and 1-and 2-year follow-up during one week.	No significant differences in exercise frequency and intensity between treatment and control children.	**Organizational:** lessons and structured PA in center **Interpersonal:** parental influence: social support **Intrapersonal:** knowledge, beliefs, attitude
Fitzgibbon et al. 2006, USA, Hip Hop to Health Jr [37]	Moderate	401 Latino children, 3-5 yr, 12 Head Start centers	Cluster RCT, unit: preschool *(SCT, SDT and TTM)*	BMI	40 min three times weekly for 14 week; 20-min lesson and 20-min physical activity component plus parent weekly newsletters.	Parents assessments of children' PA frequency and intensity pre-, post- and 1-and 2-year follow-up during one week.	No differences between groups.	**Organizational:** lessons and structured PA **Interpersonal:** parental influence: social support **Intrapersonal:** knowledge, beliefs, attitude
Fitzgibbon et al. 2011, USA, Hip Hop to Health Jr. [29]	High	618 children, 3-5 yr, 18 Chigaco Public Schools	Cluster RCT, unit: school, teacher-delivered weight control intervention group and teacher-delivered general health control group *(SCT and SDT)*	BMI	40 min two (three) times weekly for 14 weeks; 20-min lesson and 20-min PA component plus parent weekly newsletters. Initial teacher training session 3 h, additional three in-school training sessions for the intervention teachers plus weekly discussions with intervention coordinator.	Accelerometers (ActiGraph) worn 7 d at baseline and post-intervention. Valid data ≥8h/d for ≥4 d. Epoch length 15 s, cut-points Pate et al. (2006).	Intervention schools engaged in more MVPA (difference 7.46 min/d) than children in control schools.	**Organizational:** curriculum in preschools **Interpersonal:** parental and teacher influence: social support **Intrapersonal**: knowledge, beliefs, attitude

Additional file 1 Continued								
**Study**	**Quality rating**	**Sample**	**Intervention design *(reported theory)***	**Primary outcomes**	**Description of PA component**	**PA measurements**	**Effect on PA**	**Level of influence and targeted factor**
Hannon & Brown 2008, USA [38]	Moderate	64 children, 3-5 yr, one university research-oriented preschool	Pre-post design	PA	Added additional PA equipment to the outdoor playground.	Accelerometer (ActiGraph) worn for 5 d during each outdoor time before and after equipment intervention. Epoch length 15 s, cut-points by Sirard et al. (2005) (+ OSRAC-P to assess prompts)	Increases were noted in VPA (4.7%), MPA (7.8%) and LPA (3.5%), whereas sedentary time decreased by 16 %. Consistent increases in PA were observed across all 5 d of monitoring.	**Organizational:** PA equipment in preschools
Jones et al. 2011, Australia, Jump Start [39]	Moderate	97 children, 3-5yr, two childcare centers	2-arm parallel pilot cluster RCT, unit: preschool	Movement skills and PA	20-min structured activity lessons 3 times/week for 20 weeks and unstructured activities with specific PA equipment to practice new skills in the afternoons; professional development lessons for staff (4x30min workshops)	Accelerometers (ActiGraph) worn 2 consecutive d while attending childcare. Epoch length 15 s, cut-points by Sirard et al. (2005)	Significantly greater increase in cpm during preschool day compared to control group during intervention (adjust. diff. = 110.5), but not at follow-up two weeks after.	**Organizational:** structured PA lessons **Interpersonal:** teachers influence: social support **Intrapersonal:** motor skills

Additional file 1 Continued								
**Study**	**Quality rating**	**Sample**	**Intervention design *(reported theory)***	**Primary outcomes**	**Description of PA component**	**PA measurements**	**Effect on PA**	**Level of influence and targeted factor**
Nicaise et al. 2012, USA [46]	Low	4-5 yr, one preschool	Preliminary study; two cross-sectional samples of 50 (baseline) and 57 (post intervention) children	PA	Renovation and redesign of preschool's outdoor space during the time between the baseline and post intervention.	Accelerometers (ActiGraph) worn 3 d during preschool attendance. Epoch length 15s, cut-points Sirard et al. (2005). Direct observation (OSRAC-P) 5 s-observation/ 25 s- recording intervals at least 15 min, but no more than 30 min per child. Same child was observed twice. Before and after renovation sets of measurements were both performed during the fall season.	Significant decrease in time spent sedentary (-26.5%) and increase in light PA (+11.6%) and MVPA (+14.9%) measured by OSRAC-P (p<.001; p<.001; p<.001). No significant changes observed in accelerometer-based PA.	**Organizational:** renovation of preschool's outdoor space
Parish et al. 2007, USA [41]	Moderate	21 AA children, 18-38 months, one child care center(with two classes)	Alternate day exposure to intervention, based on Mastery Motivational Climate Model and TARGET structure *(AGT)*	PA	Twelve 30-min play sessions, 4d/week for 3 week, six sessions were mastery motor skills learning led by experienced teachers who used TARGET program (task, authority, recognition, group, evaluation, and time) six control sessions were free outdoor play sessions.	Actiheart heart rate monitor, worn during sessions on 2 d by four randomly selected children, outcome measure was PAHR50 index or the time spent at > 50 % resting HR. Heart rate readings recorded once every 15 s. Only relevant data for both play conditions was identified.	Master learning session induced 15 bpm higher HR than free play (p<.05), 71 % of mastery sessions were considered VPA compared with 40% during free play.	**Organizational:** outdoor playtime **Intrapersonal:** experience of mastery and high autonomy

Additional file 1 Continued								
**Study**	**Quality rating**	**Sample**	**Intervention design *(reported theory)***	**Primary outcomes**	**Description of PA component**	**PA measurements**	**Effect on PA**	**Level of influence and targeted factor**
Puder et al. 2011 Switzer-land, Ballabeina [30]	High	652 children, 40 preschool classes, 30 preschools in areas with a high migrant population	cluster RCT, unit: classes *(SEM)*	Aerobic fitness and BMI	One school year long, culture-tailored, lifestyle intervention. Child activities: 4x45min PA session/week; 1 session in gym, remainder around or in classrooms; additional sports equipment; health promoters taught 1 PA session/week, after 4 months 2 sessions/month; PA cards to home; Ballabeina event, posters with stickers. Teachers: 2 workshops, 1 meeting and parent evenings; intervention lessons, support from health promoters. Parents: 3 parent week; brochures, PA cards; leaflets. Environment: fixed or mobile equipment were installed or provided; "movement corner"; “Ballabeina game"	Accelerometers (ActiGraph) worn 5d at baseline at the end of the intervention. Valid data ≥6h/d for ≥3d. Epoch length 15 s.	No significant increase among intervention group in cpm compared to control group, but aerobic fitness measured by shuttle run test increased (11%, p=.01) and motor agility enhanced (p=.004) compared to control .	**Organizational:** structured PA, PA equipment in preschools **Interpersonal:** teachers and parental influence: social support **Intrapersonal:** aerobic fitness, coordination skills
Reilly et al. 2006, Scotland, MAGIC [42]	Moderate	545 children, mean age 4,2 yr, 36 nursery schools	Cluster RCT, unit: school (HEM)	BMI SD score	30 min/d, 3 d/week for 24 weeks; enhanced PA program to increase activity and fundamental movement skills; parent education materials. Three training session for teachers.	Accelerometer (CCM/MTI WAM 7164) worn for 6 d at baseline and 6 months after intervention; 15 randomly selected children monitored at each site. MVPA > 3200 cpm, sedentary <1100 cpm.	Group difference was marginally significant mean value being greater in control group when modelling log transformed % time in MVPA (p=.05)	**Organizational:** structured PA in nurseries and home resource pack **Interpersonal:** parental and teacher influence: social support **Intrapersonal:** knowledge, beliefs, attitude, motor skills

Additional file 1 Continued								
**Study**	**Quality rating**	**Sample**	**Intervention design *(reported theory)***	**Primary outcomes**	**Description of PA component**	**PA measurements**	**Effect on PA**	**Level of influence and targeted factor**
Sharma et al 2011, USA [43]	Moderate	75 children, 3-5 yr, two Head Start centers, four classes	Pilot-pre-post design *(SCT)*	Dietary intake and PA	Over 120 physical activities for teachers to use, parent tip-sheets complementing classroom lesson plans and physical activities, and teacher training conducted by CATCH trainers. 6-wk schedule. Classroom lessons two times per week, physical activities (10-30 min) every day.	System for Observing Fitness Instruction Time for Preschoolers (SOFIT-P). Children were observed only at active time points during the school time.	No significant changes in physical activity levels during active times at the center in participating children	**Organizational:** curriculum of child care setting **Interpersonal:** parental and teachers influence: social support **Intrapersonal:** knowledge, skills, self-efficacy
Trost et al 2008, USA [31]	High	42 children, 3-5 yr, one pre-school with four classes (2 a.m. and 2 p.m.)	Cluster RCT, unit: classes	PA	3 h teacher training to integrate PA into the curriculum, goal was to provide two 10-min lessons per session (morning/afternoon) for 8 weeks.	Accelerometer (ActiGraph) worn during preschool attendance. PA measured 2 weeks prior to start then weekly throughout the 8-week program. Epoch length 15 s, cut-points by Sirard et al. (2005). Children were observed with OSRAP for 15 min and 30 s observation epochs.	Outside + classroom MVPA was greater for intervention group for weeks 7-8 than control group ($p<.05$); intervention group had higher classroom MVPA during weeks 5-6 and weeks 7-8 ($p<.05$).	**Organizational:** integrated PA in curriculum of preschool **Interpersonal:** teacher influence: social support

Additional file 1 Continued								
**Study**	**Quality rating**	**Sample**	**Intervention design *(reported theory)***	**Primary outcome**	**Description of PA component**	**PA measurements**	**Effect of PA**	**Level of influence and targeted factor**
Winter & Sass 2011, USA, Healthy & Ready to Learn [40]	Moderate	405 children, 3-5 yr, 4 Head Start centers	Quasi-experimental, pretest-posttest *(SEM and SCT)*	BMI, motor development (Brigance) and language development (PPVT-III)	Intervention length 24 weeks. Child activities: parallel curricula at home and at school: children's literature and corresponding, complementary activities. Child activity packets: Books and all materials needed for parents and teachers to engage the child. Equipment, materials, and guidance strategies to facilitate children's participation in fun, play-based physical activities aiming to increase children's MVPA to 60 min, daily. Media-based parent training: motivating parents to engage in health promotion behaviors by implementing the child activities at home. 20 hours face-to-face and media-based teacher training.	System for Observing Fitness Instruction (SOFIT) used during guided PA sessions. 10 s-observation/10 s-recording intervals	No significant differences in MVPA during guided PA lessons compared to control group due to the small sample size and less statistical power (p=0.081), but SOFIT produced the largest effect size in this study (d=0.51).	**Organizational:** parallel PA curriculum across home and preschool context; **Interpersonal:** parental and teachers influence: social support **Intrapersonal:** knowledge, beliefs, attitude, motor skills
Van Cauwenberghe et al. 2012, Belgium [47]	Low	128 children, 4-6 yr, 4 pre-schools	Pilot within-subject design	PA	Lowering preschool playground density during recess. First two d in usual conditions, during the fourth and fifth d number of preschool classes normally sharing the playground during recess was divided into two.	Accelerometer (ActiGraph) worn during one preschool week. Valid data ≥8h/d. Epoch length 15 s, cut-points by Van Cauwenberghe et al. (2011)	Significant decrease in sedentary time (5,1%) and increase in LMVPA (+5,1%) and MVPA (+4,8%) during recess, and significant increase in MVPA (+3,8 min) during entire d (p<.001; p<.001;p<.001;p<.05, respectively).	**Organizational:** lowering playground density in preschool
Notes: PA = physical activity; RCT = randomized control trial; SES = socio-economic status; MVPA = moderate to vigorous physical activity; VPA = vigorous physical activity; AA = African-American; SCT = Social Cognitive theory; BMI = body mass index; OSRAC-P = Observation System for Recording Physical Activity in Children - Preschool; CT = control trial; SDT = Self-determination theory; TTM = Transtheoretical model; cpm = counts per minute, AGT = Achievement goal theory; HR = heart rate; HEM = Health education model; OSRAP = Observational System for Recording Activity in Preschoolers; LMVPA = light to vigorous physical activity								
